# Multimodal fiber probe for simultaneous mid-infrared and Raman spectroscopy

**DOI:** 10.1038/s41598-024-57539-4

**Published:** 2024-03-28

**Authors:** Alexander Novikov, Stanislav Perevoschikov, Iskander Usenov, Tatiana Sakharova, Viacheslav Artyushenko, Andrey Bogomolov

**Affiliations:** 1 Art Photonics GmbH, Rudower Chaussee 46, 12489 Berlin, Germany; 2 Technische Universität Berlin, Straße Des 17. Juni 135, 10623 Berlin, Germany; 3 Skolkovo Institute of Science and Technology, Bolshoy Boulevard 30, Bld. 1, 121205 Moscow, Russia; 4 Samara State Technical University, Molodogvardeyskaya Str. 244, 443100 Samara, Russia

**Keywords:** Infrared spectroscopy, Raman spectroscopy, Fibre optics and optical communications, Optical sensors

## Abstract

A fiber probe has been developed that enables simultaneous acquisition of mid-infrared (MIR) and Raman spectra in the region of 3100–2600 cm^−1^. Multimodal measurement is based on a proposed ZrO_2_ crystal design at the tip of an attenuated total reflection (ATR) probe. Mid-infrared ATR spectra are obtained through a pair of chalcogenide infrared (CIR) fibers mounted at the base of the crystal. The probe enables both excitation and acquisition of a weak Raman signal from a portion of the sample in front of the crystal using an additional pair of silica fibers located in a plane perpendicular to the CIR fibers. The advantages of combining MIR and Raman spectra in a single probe have been discussed.

## Introduction

Chemical analysis is becoming more instrumental with an increasing role of spectroscopic techniques. Given the growing complexity of tasks and samples, single spectroscopic methods may not be informative or accurate enough to achieve the desired goal of quantitative or qualitative analysis.

The joint use of two or more spectroscopic techniques enhances the capabilities of analytical methods. Multimodal measurements that combine infrared (IR), Raman, fluorescence, and other spectra can be more efficient than single-method approaches. This synergistic effect of the data augmentation has been shown for a number of practical applications^1–4^. Previous works have shown improved *ex-vivo* recognition of the tumor margins using multimodal spectroscopic measurements. The combination of fluorimetry with mid-infrared (MIR)^5^ or near-infrared (NIR) spectroscopy^6^ has been used to diagnose kidney and colorectal tumors, respectively. Enhanced vibrational spectroscopy combining MIR and NIR regions has been successfully tested in the case of abdominal cancer^2^. Multimodal spectroscopy has often been proposed for bio- and medical applications including cancer detection and classification^7^, where the combination of different techniques, such as optical coherence tomography (OCT), photoacoustics (optoacoustics), fluorescence, reflectance, Raman spectroscopy, improves diagnostic performance. One of the most common methods with the greatest clinical potential is Raman spectroscopy used in combination with fluorescence or OCT modalities, which mutually compensate for the weaknesses of their counterparts^8^. In the food and pharmaceutical industries, the use of complementary methods like IR absorption and Raman spectroscopy are highly desirable due to the compositional complexity of raw materials and products^9,10^. Integrating two complex techniques into a compact scanning probe presents numerous challenges, as the need for miniaturization and efficient radiation delivery imposes extreme constraints on the system. Nevertheless, they can be integrated into multimodal endoscopes in various combinations, the effectiveness of which is confirmed by clinical studies demonstrating the significant increase in the sensitivity, accuracy, and specificity of tumor diagnosis^7^.

The combination of two (or more) spectral methods in a single probe is always practicable and handy. Compared to probes that use only one method, it saves space and necessary sample volume, requires only a single connection port for an in-line measurement, can be manipulated with one hand, and demands less cleaning and maintenance effort. The most essential advantage of the multimodal probes is the possibility of simultaneous measurement at the same sample point. In some situations, it is critical for the analysis results. A multimodal probe is required for consistent spatial mapping in solid heterogeneous samples, such as biological tissue. In liquid samples, measurement at the same point can be critical in rapidly changing environment, such as streams or turbulent media, as in real-time process monitoring^11,12^. In both cases, even simultaneous measurements in different points cannot guarantee that chemically identical portions of analyte are observed, as required.

Combining different spectroscopic techniques within the same probe is facilitated by the application of fiber optics. An example of such a development is a compact fiber optic probe for simultaneous measurement of reflectance, fluorescence and Raman spectra of a small area (2 mm in diameter) of biological tissue^13^. This fiber probe was used in-vivo for the detection of vulnerable or thrombotic plaques during carotid endarterectomy or femoral bypass surgery^14^. Multimodal spectral probes combining fluorescence, reflectance, and Raman spectroscopy have been developed by several groups and companies^15–25^. Advances in the development of chalcogenide (CIR) and polycrystalline infrared (PIR) fiber materials expand the analytical spectral range of modern probes towards MIR^26–28^. Therefore, modern fiber spectroscopy almost seamlessly covers the entire wavelength range of optical analysis from 0.2 to 17 microns.

A recently developed multimodal probe combining attenuated total reflection (ATR) MIR and fluorescence spectroscopy has shown its efficiency in the classification of biological tissues^29^. To the best of our knowledge, this is the first and only fiber optic probe capable of augmenting the MIR-measurement with another spectroscopic data from the same location. The method combination was based on the proposed frustum design of the ATR crystal head that enables laser excitation of the medium in front of the probe and subsequent fluorescence signal detection using a built-in pair of silica fibers^30^. Another pair of CIR fibers is used to acquire the infrared spectra of nearly the same sample point as in the standard ATR probe.

The present study investigates the feasibility of obtaining the Raman spectra through the silica fiber channels using the same or a similar multimodal probe design. Despite the fact that the physical nature of fluorescence and Raman scattering is different, the same silica fibers can be used in fiber optic tools for both spectroscopic techniques. The fluorescence is related to the linear absorption of photon energy by electrons, with emission wavelengths depending on electron energy levels. Therefore, fluorescence occurs in most cases in the ultraviolet and visible range. In contrast, Raman scattering is a non-linear effect where the scattered radiation depends on vibrational energy levels. Raman scattering occurs irrespective of the excitation wavelength and can, in principle, be observed in any part of the spectrum, although the NIR region is most commonly used to avoid fluorescence. Nevertheless, fluorescence emission and Raman scattering are often observed together. Their spectral signals are typically observed in the visible and short-wave NIR radiation range^31^ and can be considered competitive in that sense. Spectroscopists from the respective communities often consider Raman signals as unwanted artifacts in fluorescence spectra, and vice versa. At the same time, the Raman effect brings a lot of structural molecular information with the uniqueness of the spectral fingerprint and band intensities related to concentration.

MIR absorption and Raman spectra carry complementary information about molecule vibrations. MIR absorption bands generally exhibit higher intensity for anti-symmetric vibrations than the respective Raman signals of the same functional group, and vice versa^32^. In spite of the affinity of chemical information provided by these two methods, their joint use in analysis is technically complicated. In fact, MIR and Raman spectral measurements of the same sample would require two different toolsets, including spectrometers and measurement interfaces^33^. The very different signal strengths of the methods, which use very different energy regions, require individual light sources, detection systems, optics, sampling techniques, etc.

A very important step towards the coupling of MIR and Raman techniques in one instrument is presented in recently published research^34^. In this work, the optical differences between methods were overcome by using a NIR femtosecond laser as a light source and a non-linear crystal which converts NIR pulses to MIR pulses. The generated MIR and non-depleted NIR pulses collinearly irradiate the sample and then they are separated after passing through the sample and simultaneously detected. The feasibility of simultaneous MIR and Raman analysis by the presented prototype has been illustrated using a few samples of pure organic liquids. The performed literature research has not revealed any attempt to create a universal measurement interface, e.g. fiber optic probe, compatible with both MIR and Raman spectral measurement, which is another very important issue of combining both methods in one analytical device.

This proof-of-concept study is aimed at obtaining a Raman spectrum through a ZrO_2_ crystal head of the recently developed multimodal ATR probe with two built-in silica-fiber channels^29^. Due to the expected optical losses in the fibers, the ATR-crystal, and the sample medium, the detection of the intrinsically weak Raman signal on any chemically informative spectral range is an experimental challenge. The proposed multimodal probe should be a useful tool accelerating the further development of complementary vibrational analysis.

## Materials and methods

**Figure 1:**
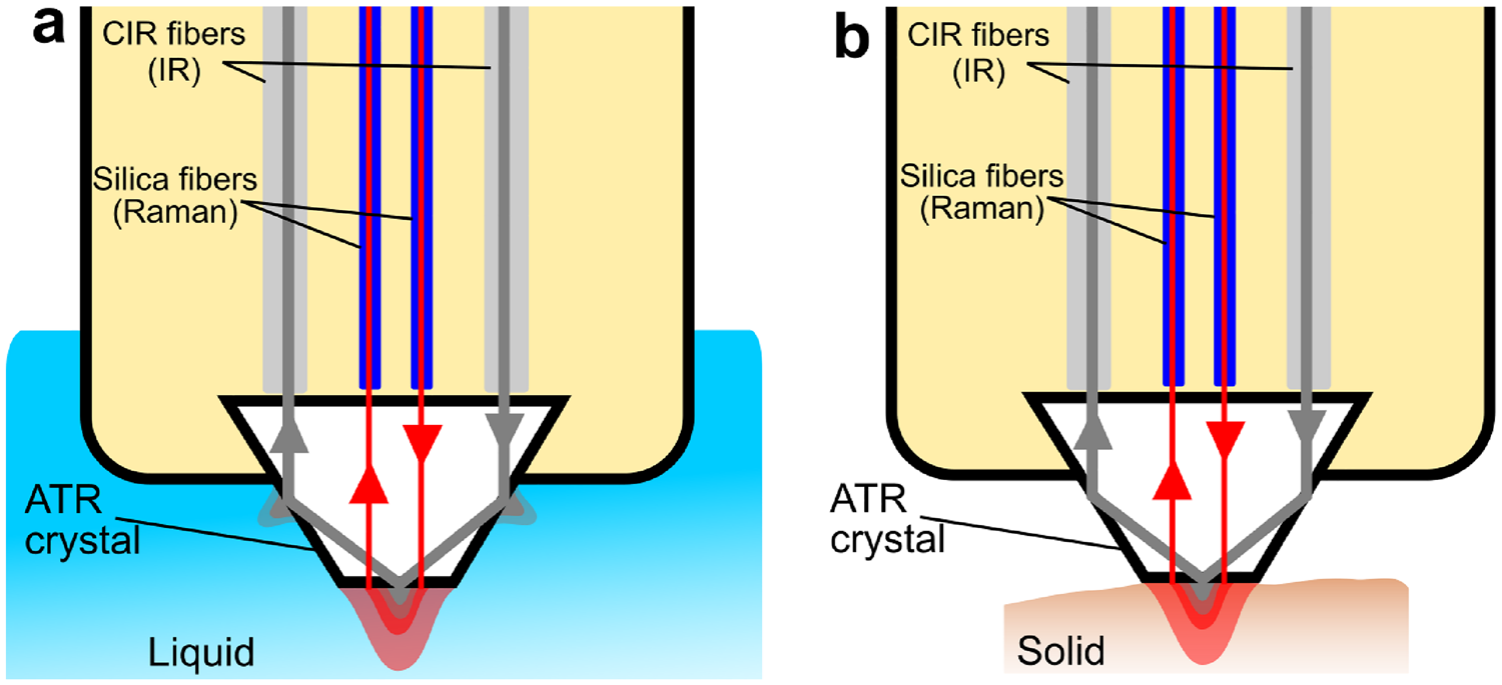
Multimodal fiber optic probe scheme with (**a**) liquid and (**b**) solid sample.

All multimodal measurements were performed through the same probe, as described in our previous work^29^. The probe's shaft is made of polyether ether ketone (PEEK) and has a diameter of 6.3 mm. The probe head has a crystal made of cubic zirconia ZrO_2_ (the cubic crystalline form of zirconium dioxide), which enables ATR-measurements with triple reflections due to its frustum shape (Fig. 1). Two CIR fibers attached to the base of the crystal were responsible for conducting the MIR radiation to/from the sample. These construction elements and the design are standard for an ATR probe of this kind. The resulting measurement range of the MIR part of the probe is limited by the transmission properties of CIR glass (1–6 µm) and ZrO_2_ crystal (0.26–7.5 µm). The incident MIR radiation is directed at an angle of 60° to the axis perpendicular to the ATR crystal surface. The same angles of incidence are observed for all reflections inside the ATR crystal^29^. At this angle, the theoretical penetration depths for liquid samples such as water, isopropanol, acetone, and cyclohexane with a refractive index of approximately 1.3 were calculated to be, for instance, 0.25 µm and 0.68 µm at wavelengths of 2 µm and 5 µm, respectively.

To enable multimodal measurements, the ATR probe was modified by adding a pair of fused silica fibers lying in a plane perpendicular to the ATR channels (no collimation optics was used). The high-OH silica fibers having transmission in the broad 0.25–1.2 µm range were used for the Raman spectroscopy in this work.

A separate silica-fiber Raman probe specially designed by art photonics GmbH (Germany) for obtaining high-quality Raman spectra was used as a benchmark. This probe was developed to work with 680-nm and 785-nm laser excitation wavelengths due to the utilized single-band bandpass laser filter with a transmission band of 625–792 nm. It provides the possibility to collect Raman signals in the whole range of Raman shifts from 200 cm^−1^ to 4000 cm^−1^ using standard Raman spectrometers of this type with a working range of 800–950 nm. The excitation sources used were 680-nm and 785-nm lasers, packaged in a single unit (Innovative Photonics Solutions, USA).

The FT-IR spectrometer Bruker Matrix-MF (Bruker Corporation, USA) was used to obtain MIR spectra. The data was collected in the 4000–400 cm^−1^ wavenumber range with a resolution of 1 cm^−1^ and 64 scans to be averaged.

Raman spectra were recorded using the WP 785 Raman Spectrometer, model WP-785-C-SR-S (Wasatch Photonics, USA) with a slit width of 50 µm, a working range of ca. 800–930 nm, and a stabilized dual-wavelengths excitation laser. The spectral ranges in Raman shift units were 2237–3967 cm^−1^ nm and 270–2000 cm^−1^, as corresponds to the excitation at 680 nm and 785 nm.

Data evaluation and visualization were performed using an on-line chemometrics software TPT-cloud (Global Modelling, Germany and Mestrelab Research, Spain)^35^.

Distilled water (Walter Schmidt Chemie GmbH, Germany), acetone (99.5%, Höfer Chemie GmbH), ethanol (80%, Carl Roth GmbH + Co. KG, Germany), and cyclohexane (99.9%, Carl Roth GmbH + Co. KG, Germany) were chosen as non-fluorescent samples to get pure Raman signals for an uncomplicated comparison of the developed multimodal probe with a standard Raman fiber optic probe.

The optimal experimental conditions were chosen by adjusting the laser power and acquisition time. For the measurements with the multimodal probe, the maximum laser power was used to illuminate the sample through the ATR crystal: about 200 mW for the 680-nm laser and 100 mW for the 785-nm laser (measured in front of the crystal tip), see Fig. S1 in Supplementary Information (SI). For the standard Raman probe the output laser power after the probe was about 220 mW for both 680-nm and 785-nm lasers. For the multimodal probe, the acquisition time was 30 ms with 1000 repetitions. The laser radiation coming out of the probe is unfocused and has a divergence corresponding to numerical aperture NA = 0.22 of silica fibers. It is worth noting that such high laser power used for the probe evaluation purposes can be dangerous, especially for solid and tissue samples. It should be reduced in the future optimized versions of the probe.

**Figure 2:**
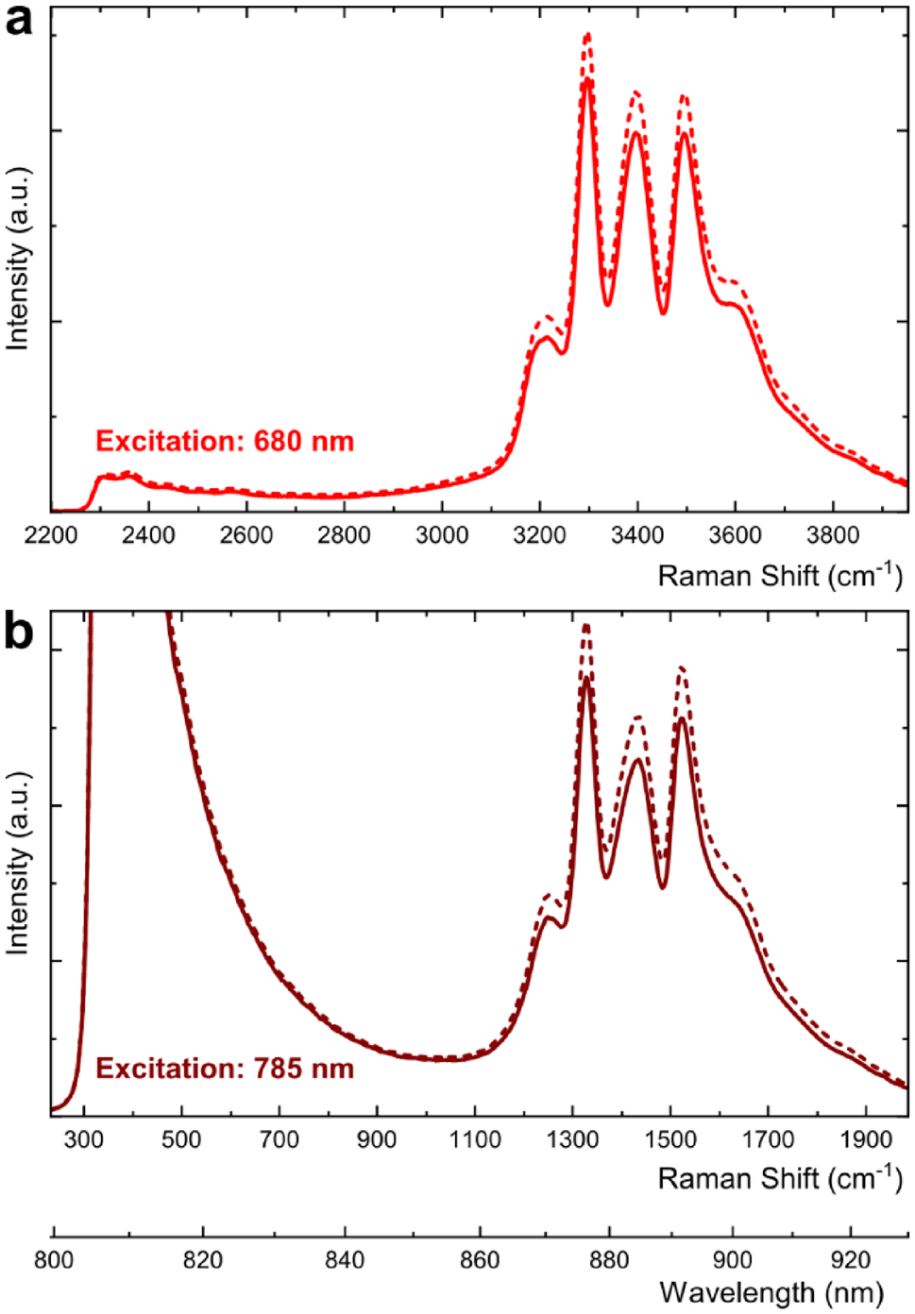
Raw Raman spectra collected with the multimodal probe in an empty vial (dashed lines) and in a vial filled with cyclohexane (solid lines) at (**a**) 680 nm and (**b**) 785 nm excitation wavelengths.

The typical raw spectra obtained with the multimodal probe are presented in Fig. 2. To eliminate the unwanted background caused by the luminescence of the ATR crystal, linear subtraction of the background spectrum (probe in air without sample) was used:

1 $$ I = I_{sample} {-} \, k \cdot I_{background} , $$

where *I, I*_*sample*_*, I*_*background*_ were the processed spectrum, sample spectrum, and background spectrum measured in air, respectively. The coefficient *k* (or subtraction factor) was selected manually using an exhaustive search with the step of 0.001 to obtain optimal spectrum shape. The direct subtraction with *k* = 1 does not eliminate the background signal completely, since the difference in refractive indices of crystal-air and crystal-liquid interfaces results in a difference in the total amount of the detected radiation.

To remove the sloping background remaining after spectral subtraction (as per Eq. (1)), an asymmetric least squares (AsLS) baseline correction algorithm^36,37^ was applied.

The emission signal at about 400 cm^−1^ (Fig. 2b) represents residual laser radiation above the upper border of the longpass filter in the Raman spectrometer (about 808 nm, as corresponds to Raman shifts of 360 cm^−1^ and 2330 cm^−1^ for 785 nm and 680 nm excitation wavelengths, respectively).

## Results and discussion

Preliminary Raman measurements using the multimodal probe (Fig. 2) have revealed a group of three very intensive peaks clearly observed in the region 862–925 nm (3100–3900 cm^−1^). The emission signal was present in all obtained spectra, including the liquid samples and the background spectrum in air (Fig. S2 in SI). Therefore, it was assigned to luminescence of the ZrO_2_ crystal of the probe. This statement was additionally confirmed by an experiment, in which individual ZrO_2_ crystals were measured using the standard Raman probe, see Fig. S3 in SI. The high intensity of the peaks and their constant positions (in the wavelength scale) that do not depend on the excitation wavelength allowed us to exclude the hypothesis that the observed effect is Raman scattering. It is known from the literature^38^ and databases^39^ that the peak positions and shapes of both spectra in Fig. 2 are characteristic of cubic zirconium oxide.

**Figure 3:**
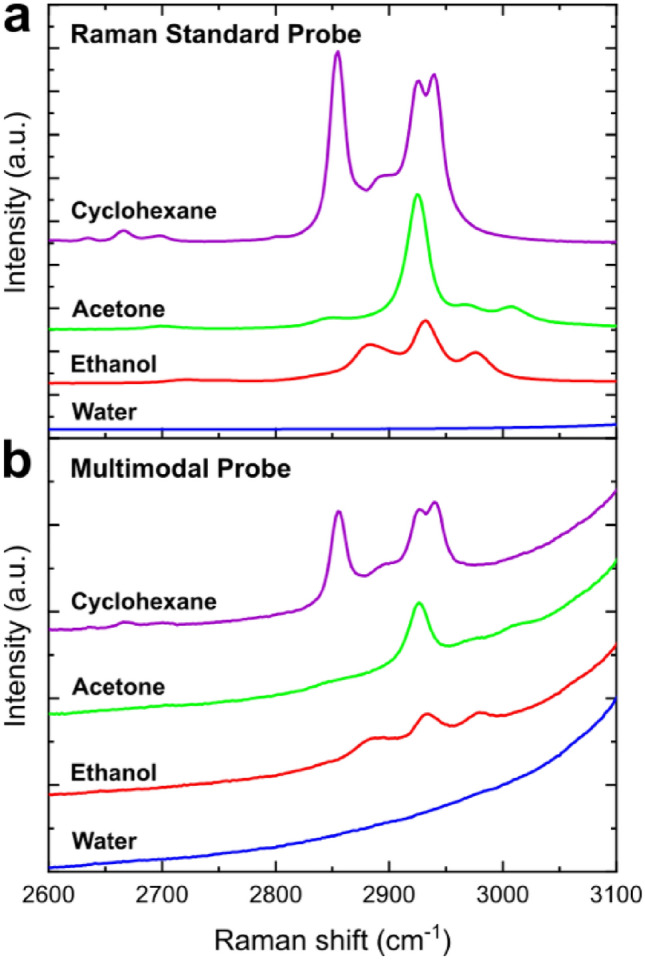
Raman spectra of pure substances (cyclohexane, acetone, ethanol, and distilled water) excited at 680 nm and collected using: (**a**) the standard Raman probe; (**b**) the multimodal probe (background spectrum subtraction was applied).

Mathematical subtraction of the background spectrum taken with a factor *k* from sample spectra (Eq. (1)) was shown to be an efficient way to reveal weak Raman signals obtained using the multimodal probe. The factor *k* was optimized to obtain a possibly simple baseline (Fig. 3b); a value of *k* = 0.823 was found to be the best for all tested samples. The value of *k* < 1 (i.e. higher spectral intensity in the air, Fig. 2) is explained by the difference in refractive indices of crystal-air and crystal-liquid interfaces, which results in a difference in the total amount of the detected radiation.

**Figure 4:**
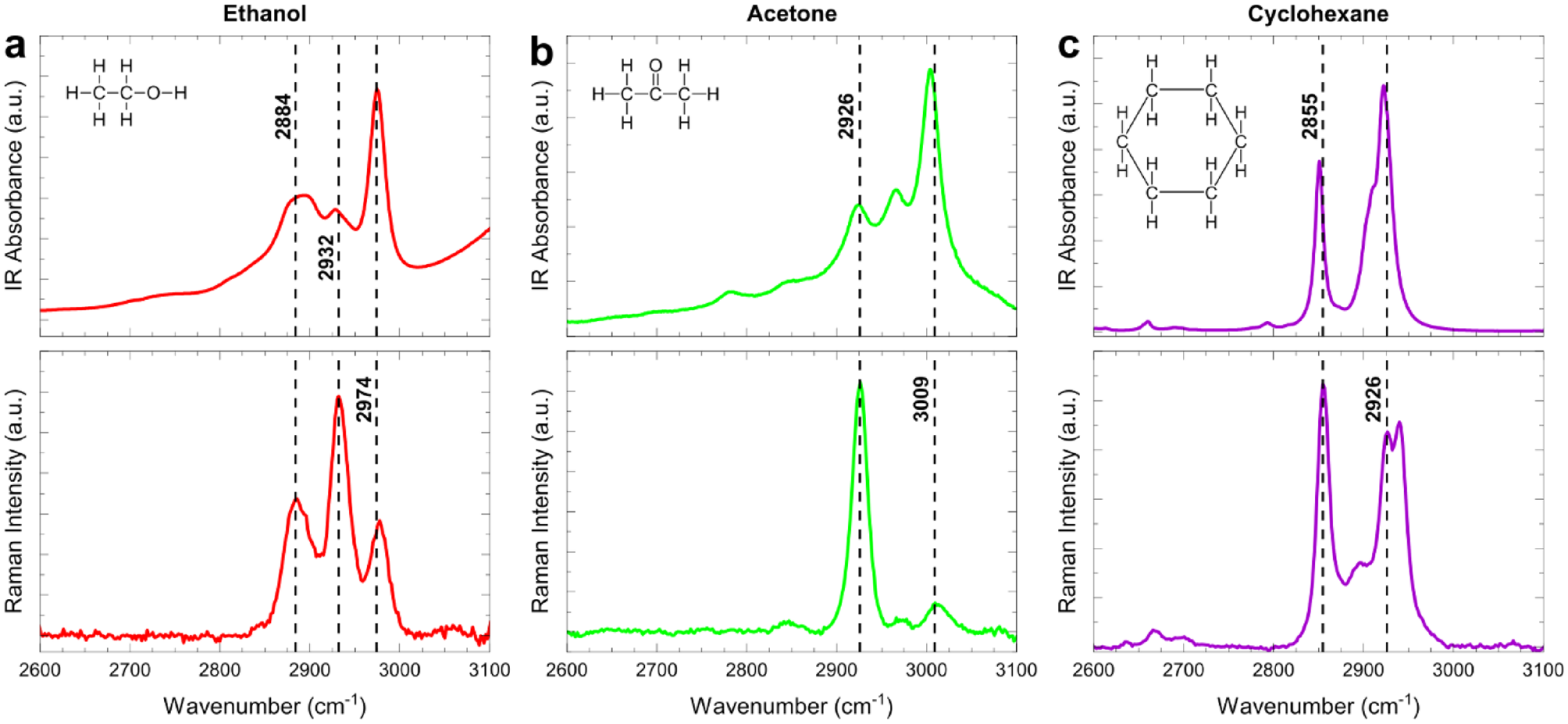
MIR and Raman spectra of (**a**) ethanol, (**b**) acetone and (**c**) cyclohexane obtained using the multimodal probe and plotted using the same frequency axis. The full-range MIR spectra are shown in SI (Fig. S5).

Although the spectral subtraction successfully eliminates the peaks of luminescence, it leaves a slope in the background that is ascending towards higher wavenumbers. This can be attributed to the dependence of the refraction properties on the wavelength and other non-linear factors influencing the observed intensity of ZrO_2_ luminescence. Hopefully, the slope can be technically compensated for in future improved versions of the probe, since it is absent in the standard probe. In the present case, however, the slope has been successfully removed using an automated baseline correction algorithm. After the baseline subtraction (Fig. 4), the spectra in the studied region look very similar to those obtained with the standard Raman probe (Fig. 3a).

Full Raman spectra of the three pure organic solvents (cyclohexane, ethanol, and acetone) and water, taken with the standard probe at two different excitation wavelengths (680 and 785 nm), are presented in Fig. S4 (SI). As it can be seen, the two spectral intervals (200–2000 cm^−1^ and 2200–4000 cm^−1^) cover almost the entire region of 200–4000 cm^−1^ and contain the most relevant vibrational information about the functional groups of the molecules being studied.

The same spectral measurements performed with the multimodal probe have produced the following results. The probe is relatively transparent to the visible light and NIR radiation in the region of 0.26–1.2 µm, which is an intersection of transmission regions of high-OH silica fibers (0.25–1.2 µm) and ZrO_2_ crystal (0.26–7.5 µm^40,41^) used to acquire the Raman spectra. Distinct Raman signals were observed in the high-wavenumber region and particularly in the range 2600–3100 cm^−1^, which corresponds to the laser excitation wavelength of 680 nm (Fig. 3). Comparing these measurements (Fig. 3b) to the identical measuring range with high-quality Raman probe (Fig. 3a), one can see the sameness of spectral shapes, which confirms the principal feasibility of Raman measurements using the multimodal probe. The observed peaks were weak and could only be seen after the subtraction of the background spectrum, as described in Sect. 2. When the spectra are obtained under optimal conditions no mathematical smoothing is required. The region of higher wavenumbers (above 3100 cm^−1^) that contains O–H bond stretching vibration signals of ethanol and water has been completely shaded by the strong luminescence of the ATR crystal head. Another adjacent region 2200–2600 cm^−1^ does not contain any useful spectral information for the selected substances.

Unfortunately, an attempt to see any Raman signal in the fingerprint region (excitation at 785 nm) has brought no positive result. The main problem preventing the measurements on particular spectral intervals is the strong luminescence from the cubic zirconia crystal. This issue is quite typical for the Raman spectroscopy. The background signal subtraction using Eq. (1) was successful in the spectra excited at 680 nm laser wavelength and observed below 862 nm (Raman shift below 3100 cm^−1^), which is less affected by the luminescence (Fig. 2a). The measurement dynamic range of the spectrometer is insufficient to observe weak Raman signals against the luminescence background that is several orders of magnitude stronger. If the spectrometer is set to a higher sensitivity, as necessary for the detection of Raman signals above 862 nm, the detector is levelled-off (Fig. S1 in SI). Therefore, the informative fingerprint region of Raman shifts (below 2000 cm^−1^), which requires the laser excitation at 785 nm (Fig. 2b), is entirely blocked in the case of the ZrO_2_ crystal element of the ATR head. At the same time, the 300–1110 cm^−1^ region in Fig. 2b contains a strong tail from the 785-nm laser peak. This is an important experimental conclusion that helps in further optimization of the multimodal probe.

As it follows from the above discussion, the main interest in terms of the complementary MIR and Raman multimodal analysis presents in our case the region of high wavenumbers and particularly the interval 2600–3100 cm^−1^. It is available for spectral observations by both methods using the multimodal probe. The respective Raman spectrum is excited at the 680 nm laser wavelength. The MIR and Raman spectra of organic solvents in the region 2600–3100 cm^−1^ acquired using the multimodal probe are plotted in Fig. 4 (MIR spectra are separately plotted in Fig. S5 in SI) using the same frequency axis to facilitate their direct comparison and spectral interpretation. Raman spectra were preprocessed to eliminate strong background effects (Sect. 2). Spectral quality in both cases is good and suitable for the data analysis.

Peak assignment of the two techniques to the functional groups (Table S1 in SI) shows that the selected region contains predominantly the responses of C-H stretching vibrations of hydrocarbon residues present in most organic substances, polymers and biological macromolecules^42^. Spectroscopic analysis in this narrow region is complicated, because signal intensities are basically moderate and the peaks strongly overlap, which cannot be avoided even in the high-resolution spectra. On the other hand, methyl (CH3-) and methylene (-CH2-) are the most widespread functional groups in organic substances. The vibrational signals in this region are sensitive to the chemical environment of the respective functional groups in the molecule, such as unsaturated bonds or electronegative atoms^43^, which may affect their fundamental frequencies and make the analysis more specific. Application of the modern data analysis techniques essentially compensates for the problem of overlapping signals in the high-wavenumber range and makes it well suited for quantitative analysis. Figure 4 illustrates the fact that only two fundamental vibrations of methyl and ethyl groups create essential spectral diversity, and thus, informativeness, which is further emphasized by the complementary usage of MIR and Raman measurements. Although the spectra obtained with the multimodal probe are observed in a technically limited spectral region, combining them in one measurement produces a synergistic effect.

**Figure 5:**
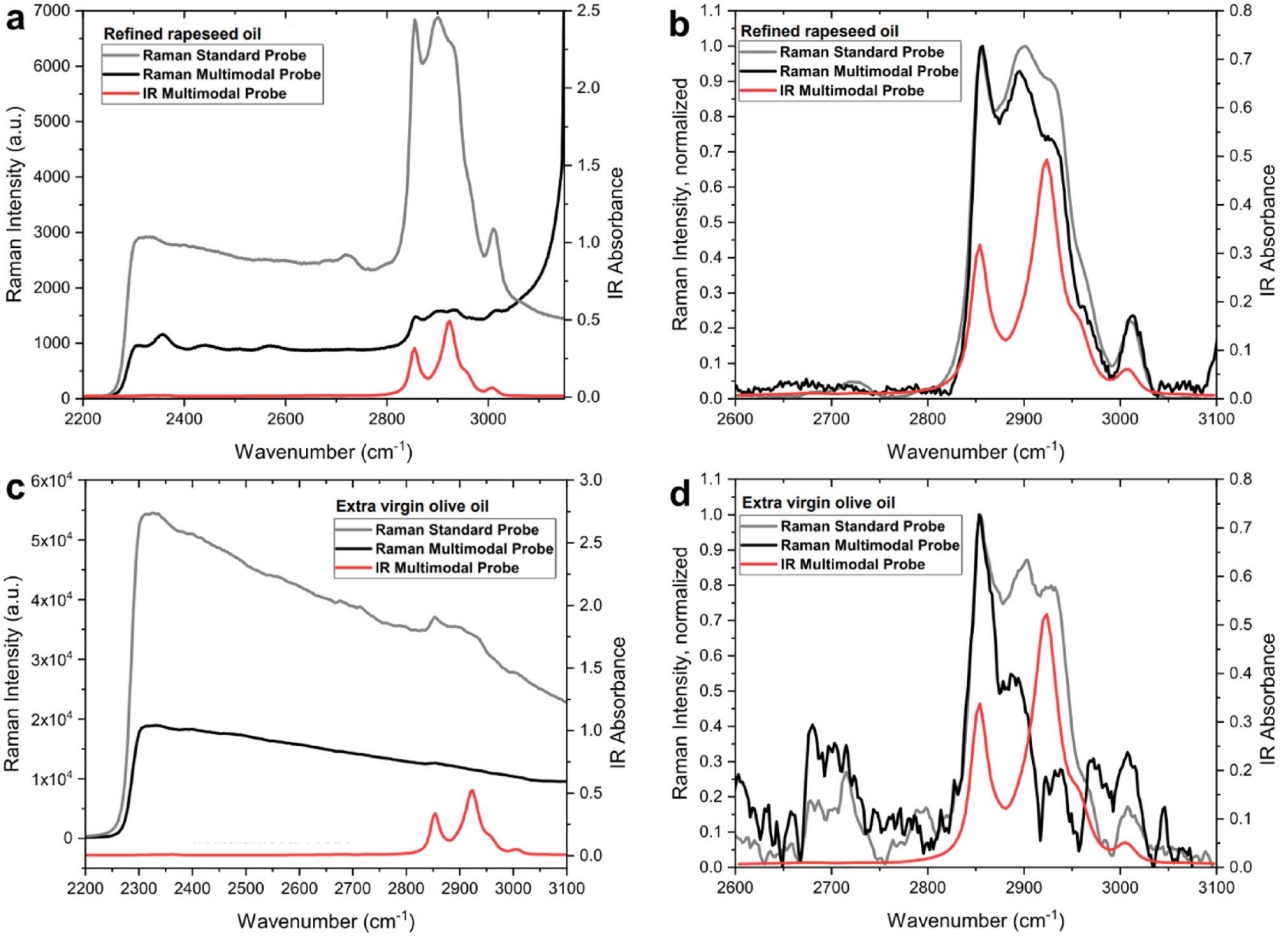
Raman (excited at the wavelength of 680 nm) and MIR spectra of (**a**,**b**) the refined rapeseed oil: (**a**) raw data, (**b**) after baseline correction of Raman spectra; and (**c**,**d**) the extra virgin olive oil: (**c**) raw data, (**d**) after baseline correction of Raman spectra.

Further evaluation of the multimodal probe was performed using two complex natural samples: extra virgin olive oil and refined rapeseed oils. The main composition of the samples is similar, but the Raman spectroscopy of extra virgin olive oil is complicated by the presence of strong fluorescence emission in the same spectral region. The higher fluorescence of the olive oil is due to the presence of minor chemical components that can be removed after refining. The corresponding Raman and MIR spectra are presented in Fig. 5. Fig. S6 in SI illustrates the laser light propagation in an oil sample observed using the multimodal probe.

The Raman signals in the spectra taken from the refined rapeseed oil and extra virgin oil with the multimodal probe and with the standard probe are also observed on a strong fluorescence background (Fig. 5a,c). Nevertheless, after applying spectral subtraction and baseline correction both probes produce Raman spectra of comparable quality for both studied oils (Fig. 5b,d). Some differences in the shape of Raman spectra obtained with the multimodal probe are due to intrinsic signal weakness and the intensive mathematical transformations applied to suppress unnecessary background. This is particularly true for highly fluorescent samples of extra virgin oil (Fig. 5c,d), where the signal-to-noise ratio is lower.

Distinguishing different oils from each other is of practical importance. It can be necessary to recognize the product adulteration, when an expensive product is diluted or replaced by a low-cost substitute^44^. Examples of this kind are shown in Fig. 5, which represents the vibrational spectra of the cheapest rapeseed and a high-grade extra virgin olive oil. Wide variability in the chemical composition of the natural products make the analytical characterization of olive oil samples difficult. Nevertheless, despite the fact that quantification of oil adulterant is challenging, Raman and MIR spectroscopy approaches have been applied for the authentication of edible oils and the detection of adulteration^45–47^. The presented MIR and Raman spectra of vegetable oils are similar, which is explained by their close chemical similarity^48,49^. This circumstance makes it difficult to distinguish them by any of these vibrational spectroscopy methods, even with the use of their combination. The fluorescence of unrefined extra virgin olive oil is due to minor components containing a system of conjugated unsaturated bonds^50^. Therefore, the fluorescence signal in this case represents an additional source of chemical information that can be used along with vibrational spectra to clearly discriminate two vegetable oils from each other. Depending on the sample we can focus on either the fluorescence or on the Raman signal applying appropriate mathematical processing to the data. This particular example perfectly illustrates the advantage of having three different spectroscopy methods in one spectral measurement. Depending on the sample nature and the analytical problem being solved, various pairwise combinations of methods, and in some cases their triple combination, can result in a synergistic improvement. Further development of the probe should take the nature of the analyzed samples into account, in order to obtain the optimal quality of the MIR, Raman and fluorescence spectra supplied by the multimodal probe.

## Conclusions

The multimodal fiber optic probe proposed and tested in this work helps to reveal the advantages of simultaneous MIR and Raman spectroscopy, which is traditionally complicated by significant technical and methodical differences between these methods. The probe allows focusing the measurements at the same point and synchronizing them in time. This is very important for the analysis of processes and heterogeneous samples.

The observed Raman signals were much weaker, compared to the standard Raman probe, and were affected by strong luminescence effects of the sample and probe itself. Nevertheless, it was possible to obtain a good quality spectrum in the region 2600–3100 cm^−1^ after simple mathematical data processing. This spectral region is particularly valuable for the complementary MIR and Raman analysis, because it reflects a lot of information about the C–H stretching vibrations of aliphatic hydrocarbons. The possibility of simultaneous acquisition of MIR and Raman spectra can significantly increase the analytical importance of this region.

The presence of fluorophores can complicate the analysis, which is a typical problem in Raman spectroscopy. It was shown, however, that in some cases the fluorescence signal can be used as an additional source of useful chemical information. Combining two or even more spectroscopic techniques in one measurement interface is a prospective way of developing instruments and methods for the analysis of complex samples. Considering the modern progress of optical multisensor systems, the triple or pairwise combinations of the above three methods of spectroscopy proposed in this work can be used for creating compact analyzers optimized for different practical applications.

Future research should be aimed at studying the capabilities of the proposed multimodal method and probe to define the most advantageous practical application for each possible combination of simultaneously used techniques. The probe design and measurement geometry should be respectively optimized. It is expected that replacing the ZrO_2_ ATR element with a less or non-luminescent material in the 700–900 nm region will lead to a significant improvement in signal strength and range enhancement of Raman spectra. Diamond crystal with a relatively narrow single Raman peak at 1332 cm^−1^^39,51^ represents a possible, albeit a more expensive, alternative material to zirconium dioxide. The mathematical signal processing algorithms proposed in the work can be further improved and automated to facilitate the analysis.

### Supplementary Information

Supplementary Information.

## Data Availability

The datasets used and/or analyzed during the current study available from the corresponding author on reasonable request.
