# Supplementary material for: Multimodal fiber probe for simultaneous mid-infrared and Raman spectroscopy
Source: Sci Rep. 2024 Mar 28;14:7430. doi: 10.1038/s41598-024-57539-4 (PMC10978856; doi:10.1038/s41598-024-57539-4)
Supplement: Supplementary file 1 — Supplementary Information. [file 41598_2024_57539_MOESM1_ESM.pdf]

Supplementary information

Multimodal fiber probe for simultaneous mid-infrared and Raman spectroscopy

Alexander Novikov,[1,2*] Stanislav Perevoschikov,[1,3] Iskander Usenov,[1,2] Tatiana Sakharova,[1] Viacheslav Artyushenko,[1] Andrey Bogomolov[1,4]

[1] *art photonics GmbH, Rudower Chaussee 46, Berlin 12489, Germany*

[2] *Technische Universität Berlin, Straße des 17. Juni 135, 10623 Berlin, Germany*

[3] *Skolkovo Institute of Science and Technology, Bolshoy Boulevard 30, bld. 1, 121205 Moscow, Russia*

[4] *Samara State Technical University, Molodogvardeyskaya 244, 443100 Samara, Russia*

[*] Corresponding author: an@artphotonics.de (Alexander Novikov)

Abstract

A fiber probe has been developed that enables simultaneous acquisition of mid-infrared (MIR) and Raman spectra in the region of 3100-2600 cm^{-1}. Multimodal measurement is based on a proposed design of a ZrO_2 crystal at the tip of an attenuated total reflection (ATR) probe. Mid-infrared ATR spectra are obtained through a pair of chalcogenide infrared (CIR) fibers mounted at the base of the crystal. The probe enables both the excitation and acquisition of the weak Raman signal of the sample portion in front of the crystal using an additional pair of silica fibers located in a plane perpendicular to the CIR fibers. The advantages of the complementary use of MIR and Raman spectra in the working region have been discussed.

Additional experimental results

The operating wavelength window of the probe was determined by the transmission of the ATR element and the fibers. Accordingly, it is necessary to select the excitation laser wavelength. The luminescence of the ATR element can also cause difficulties in sample analysis. In our case, the ATR element is made of cubic zirconia ZrO_2, which has a strong photoluminescence (Fig. S-1) which limits the operating range. In our case it limits the Raman shift axis to 3100 cm^{-1} To eliminate such problems, the material of the ATR element can be changed, for example, with diamond that have sufficiently high refractive index and good transmittance in the visible and IR regions.

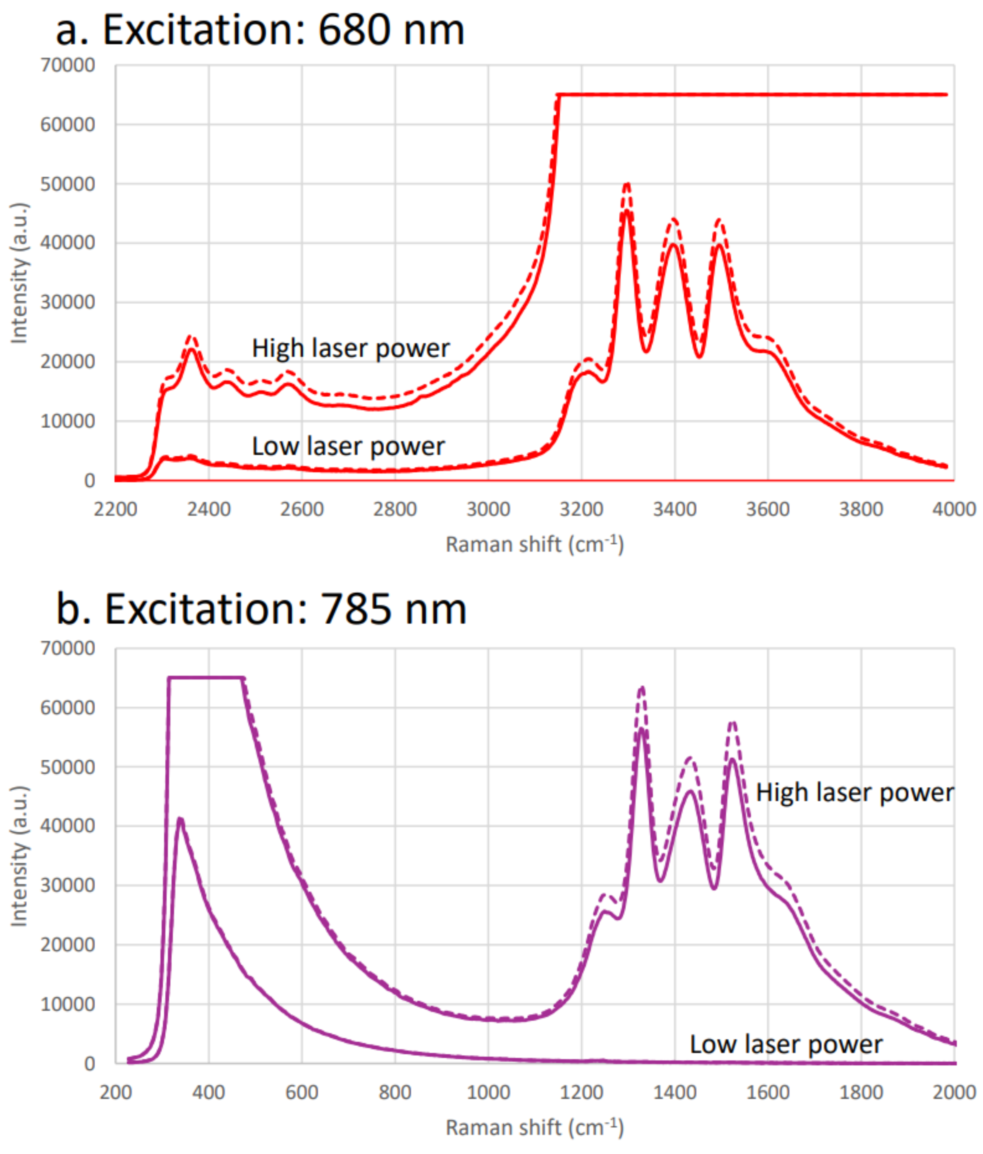

Figure S1. Raw Raman spectra collected with the multimodal probe in an empty vial (dashed lines) and in a vial filled with cyclohexane (solid lines) at (a) 680 nm and (b) 785 nm excitation wavelengths at high (with detector saturation) and optimal laser power.

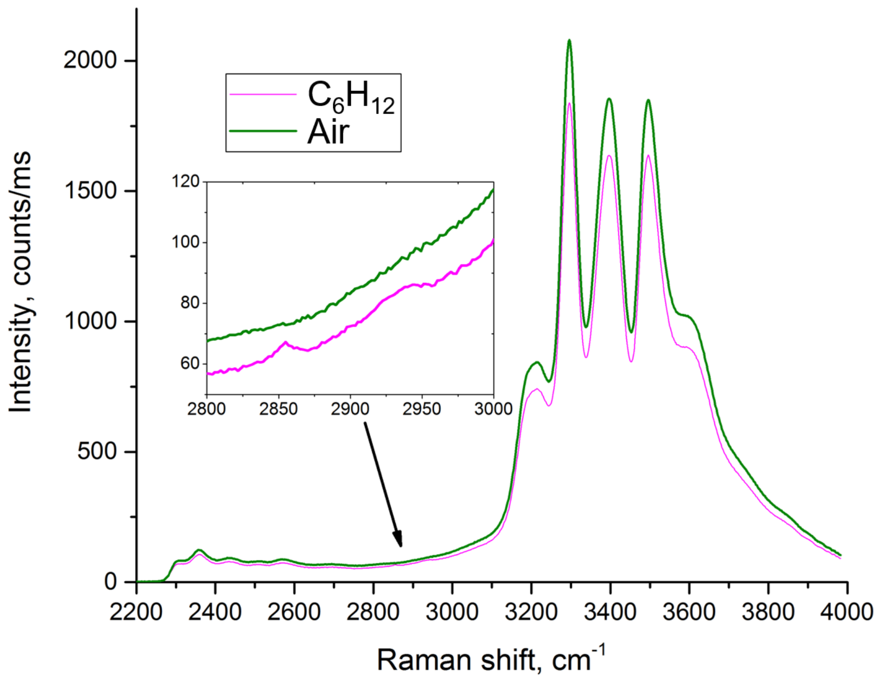

Figure S2. Raw Raman spectra collected with the multimodal probe in an empty vial (labeled “air”) and in a vial filled with cyclohexane (labeled “C_6H_{12}”) at 680 nm excitation wavelengths.

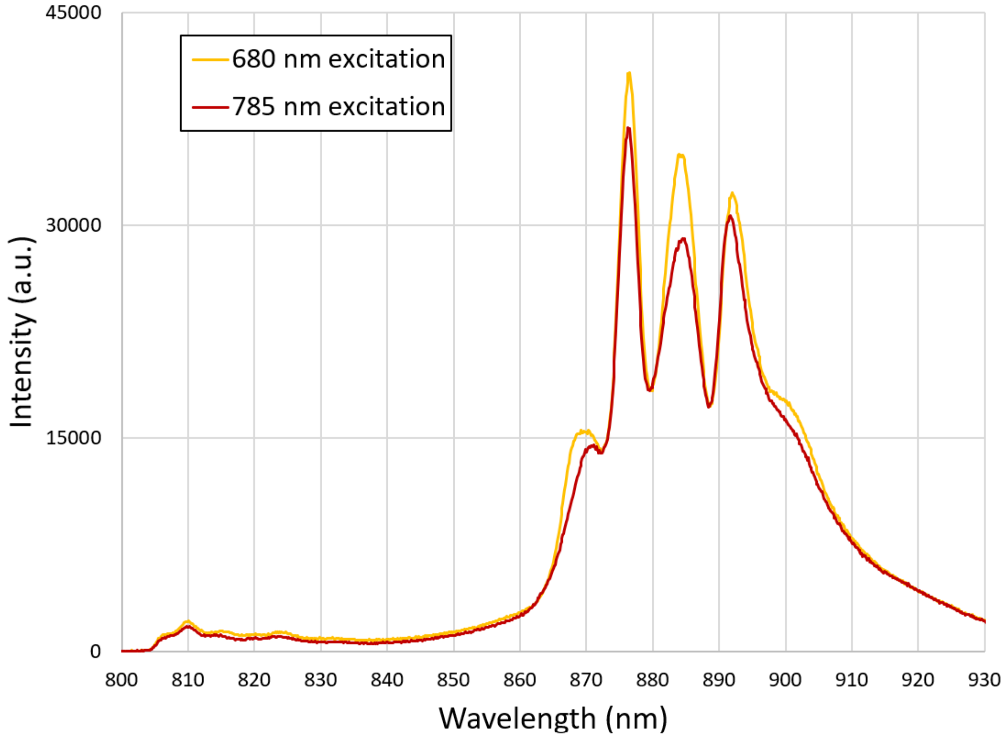

Figure S3. Spectra of ZrO_2 crystals obtained with the standard Raman probe at different excitation wavelengths.

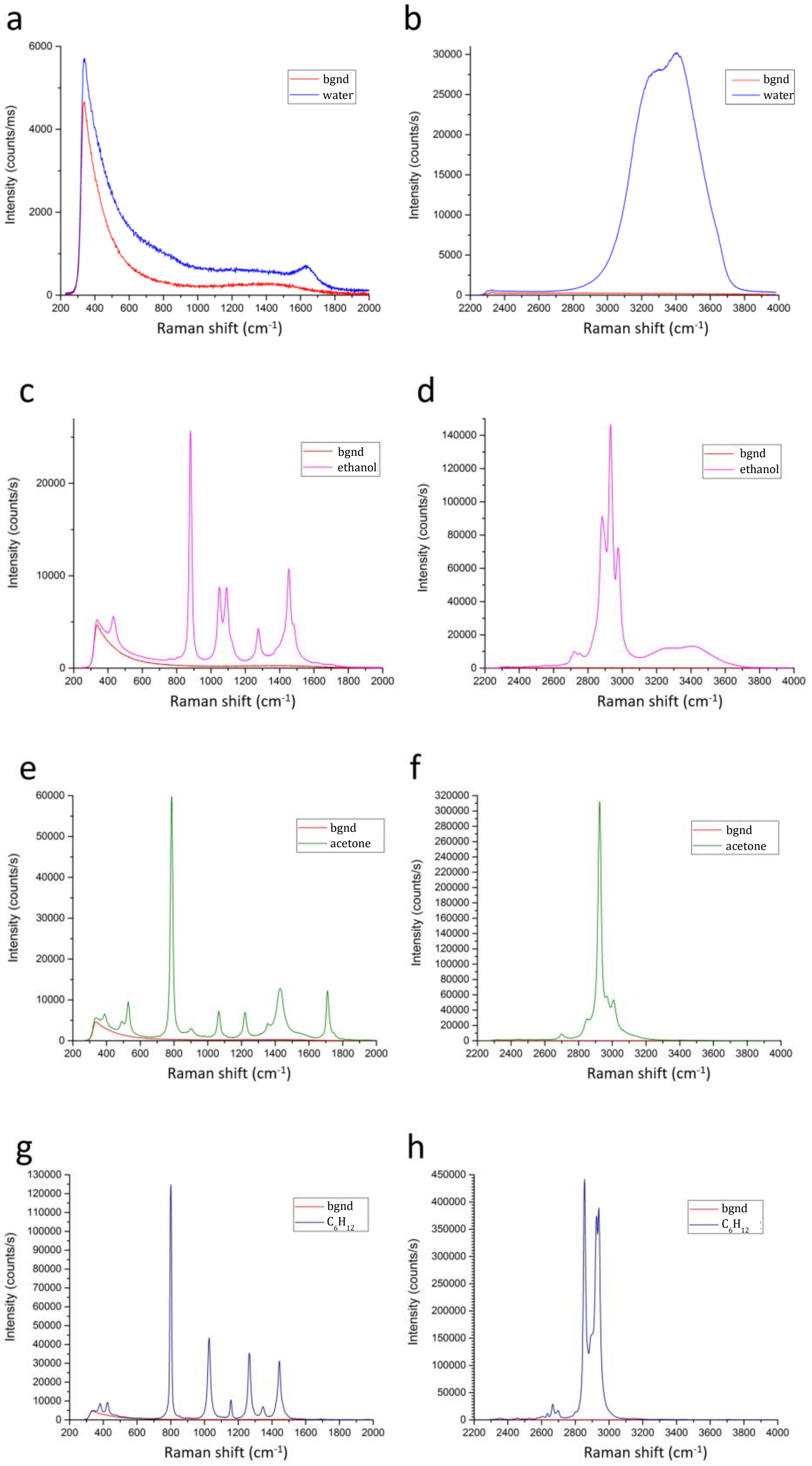

Figure S4. Raw Raman spectra of (a,b) water, (c,d) ethanol, (e,f) acetone, and (g,h) cyclohexane (labeled “C_6H_{12}“) obtained using the standard Raman probe at different excitation wavelengths: 785 nm (a,c,e,g) and 680 nm (b,d,f,h). Background spectra (labeled “bgnd”) are obtained in air (no sample).

MIR spectra of the same samples measured using the multimodal probe are presented in Fig. S-5. The working region of spectra is limited by the CIR-fiber transmission that falls down quickly below 1500 cm^{-1}. Distinct groups of peaks are observed in the accessible spectral range. This region can be useful for the analysis of organic samples including biological tissue [1]. The disadvantage of this region is related to the fact that the most of fundamental vibrations are observed at higher wavenumbers.

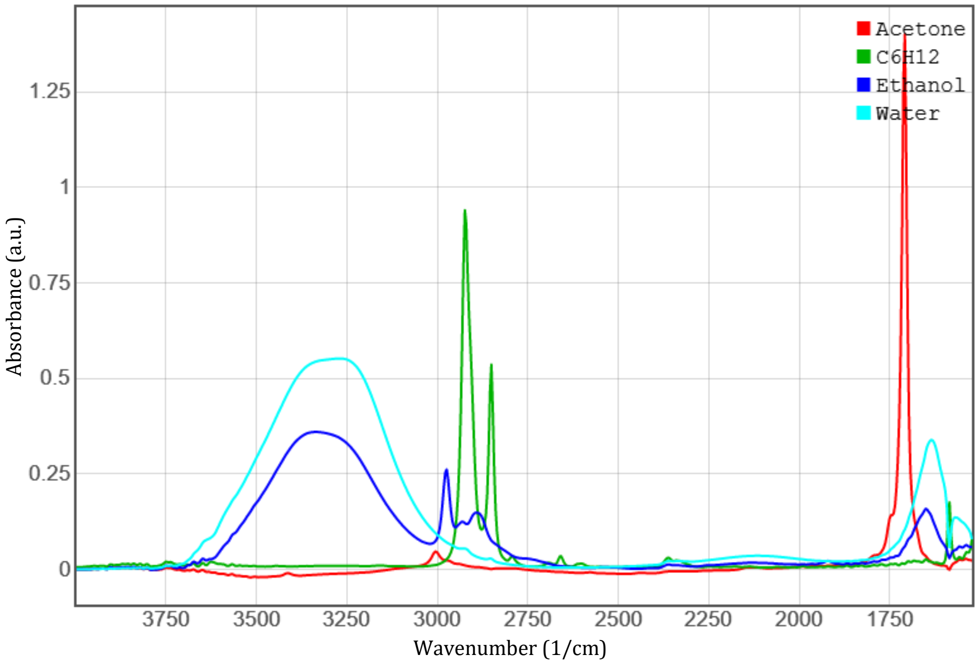

Figure S5. MIR spectra of the studied compounds obtained using the multimodal ATR probe.

Laser light beams emanating from the ATR probe can be seen visually for some samples (Fig. S-5).

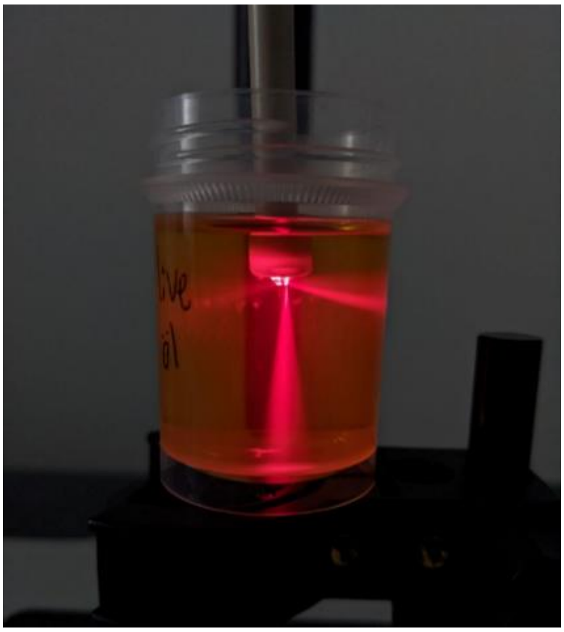

Figure S6. Laser light (680 nm) propagation in extra virgin olive oil observed using the multimodal probe.

Additional discussion

While Raman spectroscopy provides information about the vibrational energy states of a molecule, fluorescence is associated with electronic energy transitions. These two complementary spectral methods, which have their own advantages and disadvantages, can also be used together for multimodal analysis [1,2]. In general, Raman is much weaker and is not always observable against the background of fluorescence [3]. To decrease the fluorescence signal and therefore increase Raman-to-fluorescence signals ratio, lasers with longer wavelengths can be used for excitation [4]. Lasers with shorter excitation wavelengths can be also used to increase Raman-to-fluorescence ratio. It is known that intensity of Raman scattering is proportional to the fourth power of the inverse excitation wavelength [5]. The use of an ultraviolet (UV) laser will result in an amplification of the Raman signal, and the Raman peaks can be separated from the resulting fluorescence spectrum and filtered out [6].

The simplest way to increase the Raman signal intensity is to increase the number of Raman excitation and collection fibers according to the geometry of the ATR element and the spectrometer input optics. Another way to improve the probe performance would be to change the measurement geometry. Additional lenses mounted inside the probe can adjust the beam path to enhance the collected signal. A suitable longpass filter can also be built into the probe or mounted on the ends of silica fibers. A mirror at the bottom of the measurement cell A mirror at the bottom of the measurement cell can also increase the collected signal, since Raman scattering is considered an omnidirectional phenomenon. The ATR crystal shape can also be changed to provide a more efficient excitation and collection.

The presence of silica fibers in the multimodal probe also creates the potential for near-infrared (NIR) reflection analysis.

Interpretation of MIR and Raman spectra

The selected region 2600–3100 cm^{-1} contains predominantly the responses of C-H stretching vibrations of hydrocarbon residues that has been widely employed in organic, analytic, biological, and polymer chemistry [7] this region is narrow, the signal intensities are basically moderate and the data analysis is complicated by strong peak overlap, which cannot be avoided even in the high-resolution spectra. For this reason, it is generally less informative than the fingerprint region of the MIR spectrum. On the other hand, methyl (CH3-) and methylene (-CH2-) are the most widespread functional groups in organic substances. The vibrational signals in this region are sensitive to the chemical environment of the respective functional groups in the molecule that may affect their fundamental frequencies, such as unsaturated bonds or electronegative atoms [8], which makes the analysis more specific. Application of the modern data analysis techniques essentially compensates for the problem of overlapping signals in the high-wavenumber range and makes it well suited for quantitative analysis.

Raman and MIR spectra in the region 2600–3100 cm^{-1} obtained with the multimodal probe are presented in Fig. 4 of the main paper text. Raman spectra were preprocessed to eliminate strong background effects (section 2). Peak assignment of the two techniques to the functional groups is shown in Table S-1. The main peaks belong to the antisymmetric and symmetric C-H stretching vibrations of methyl and ethyl groups. Relative intensities of the peaks correlate with two factors: the abundance of the corresponding group in the molecule and its MIR and Raman spectral activity, which is determined by symmetry of a molecule. For a molecule to be MIR active, it must undergo a change in dipole moment during vibration. For a molecule to be Raman active, it must undergo a change in polarizability during a vibrational transition. In general, symmetric vibrations and non-polar groups have weak MIR-signal and are easily studied by Raman and *vice versa*: antisymmetric and polar groups are easily studied by MIR technique.

The highest general intensity in both MIR and Raman spectra belongs to cyclohexane, because the -CH2- group occurs six times in the molecule and both symmetric and antisymmetric C-H vibrations are active in the

vibrational spectrum. Ethanol is represented by the same three peaks having close positions and different intensities in MIR and Raman spectra. Antisymmetric stretching vibrations of the same group tend to have higher relative intensity in MIR spectra than in Raman and *vice versa*. Electronegative substituents result in a noticeable peak shift towards higher wavenumbers. Therefore, wavenumbers of observable peaks positions of -CH2- increase from cyclohexane to ethanol. Similarly, observable peak positions for CH3- of acetone have higher wavenumbers than those of ethanol [8]. These observations illustrate the fact that only two fundamental vibrations of two common functional groups (methyl and ethyl) create essential spectral diversity, and thus, informativeness, which is further emphasized by the complementary usage of MIR and Raman measurements. Spectral quality in both cases is good and suitable for qualitative and quantitative analysis.

Table S-1. Peak assignment to functional groups. General regions, symmetries of stretching modes and peak intensities in MIR and Raman spectra (Int MIR and Int Ram) are given in accordance with [8] where m is medium, ms is medium-strong, wm is weak-medium, asym and sym stands for antisymmetric and symmetric, respectively.

**Functional group**	**C-H stretching mode**	**General region, cm^{-1}**	**Int MIR**	**Int Ram**	**Observed peaks MIR, cm^{-1}**	**Observed peaks Raman, cm^{-1}**
$-CH_2-$ in cyclohexane	sym	2870-2840	m	ms	2850 (2853)*	2855 (2853)
	asym	2940-2915	ms	ms	2924 (2928)	2926 (2923)
$-CH_2-$ in ethanol	sym	2890-2850	m	ms	2889 (2887)	2884 (2878)
	asym	2970-2920	ms	m	2974 (2974)	2978 (2973)
CH_3- in ethanol	sym	2940-2860	m	ms	2931 (2927)	2932 (2927)
	asym	2990-2960 [9]	ms	m	2974 (2973)	2974 (2974)
CH_3- in acetone	sym	2970-2840	m	ms	2925 (2926)	2926 (2921)
	asym	3045-2965	ms	wm	3005 (3005)	3009 (3002)

Notes: * in brackets - value from Spectral Database for Organic Compounds [10])

References

1. Bogomolov, A. *et al.* Fiber probe for simultaneous mid-infrared and fluorescence spectroscopic analysis. *Analytical Chemistry* **93**, 6013-6018. https://doi.org/10.1021/acs.analchem.1c00080 (2021).
2. Xiong, H. *et al.* Stimulated Raman excited fluorescence spectroscopy and imaging. *Nature photonics* **13**, 412-417. https://doi.org/10.1038/s41566-019-0396-4 (2019).
3. Wei, D., Chen, S., & Liu, Q. Review of fluorescence suppression techniques in Raman spectroscopy. *Applied Spectroscopy Reviews* ***50***, 387-406. https://doi.org/10.1080/05704928.2014.999936 (2015).
4. Hanlon, E. B. *et al.* Prospects for in vivo Raman spectroscopy. *Physics in Medicine & Biology* **45**, R1. https://doi.org/10.1088/0031-9155/45/2/201 (2000).
5. Smith, E., & Dent, G. Modern Raman spectroscopy: a practical approach. John Wiley & Sons (2019).
6. Song, L. M. W. K., & Marcon, N. E. Fluorescence and Raman spectroscopy. *Gastrointestinal Endoscopy Clinics* **13**, 279-296. https://doi.org/10.1016/S1052-5157(03)00013-8 (2003).
7. Yu, Y. *et al.* New C− H stretching vibrational spectral features in the Raman spectra of gaseous and liquid ethanol. *The Journal of Physical Chemistry C* **111**, 8971-8978. https://doi.org/10.1021/jp0675781 (2007).
8. Socrates, G. Infrared and Raman characteristic group frequencies: tables and charts. John Wiley & Sons (2004).

9. Konaka, S., Hirose, J., Suwa, A., Takeuchi, H., Egawa, T., Siam, K., ... & Schäfer, L. Structural investigation of t-butylethylamine by gas electron diffraction, ab initio calculations and vibrational spectroscopy. *Journal of molecular structure* **244**, 1-16. https://doi.org/10.1016/0022-2860(91)80143-R (1991).
10. Spectral Database for Organic Compounds, SDBSWeb: https://sdbs.db.aist.go.jp, National Institute of Advanced Industrial Science and Technology (Accessed 03.10.2021).
